# Energy transmission through radiative ternary nanofluid flow with exponential heat source/sink across an inclined permeable cylinder/plate: numerical computing

**DOI:** 10.1038/s41598-023-49481-8

**Published:** 2023-12-14

**Authors:** Muhammad Bilal, Muhammad Waqas, Jana Shafi, Mati ur Rahman, Sayed M. Eldin, Mohammed Kbiri Alaoui

**Affiliations:** 1 Department of Mathematics, Sheikh Taimur Academic Block-II, University of Peshawar, Peshawar, 25120 Khyber Pakhtunkhwa Pakistan; 2 Mathematics Department, City University of Science and Information Technology, Peshawar, 25000 Pakistan; 3 Department of Computer Science, College of Arts and Science, Prince Sattam bin Abdul Aziz University, 11991 Wadi Ad-Dawasir, Saudi Arabia; 4 Department of Computer Science and Mathematics, Lebanese American University, Beirut, Lebanon; 5 School of Mathematical Sciences, Jiangsu University, Zhenjiang, 212013 China; 6 Center of Research, Faculty of Engineering, Future University in Egypt, New Cairo, 11835 Egypt; 7 Department of Mathematics, College of Science, King Khalid University, P.O. Box 9004, 61413 Abha, Saudi Arabia

**Keywords:** Energy science and technology, Mathematics and computing, Nanoscience and technology

## Abstract

The steady two-dimension (2D) ternary nanofluid (TNF) flow across an inclined permeable cylinder/plate is analyzed in the present study. The TNF flow has been examined under the consequences of heat source/sink, permeable medium and mixed convection. For the preparation of TNF, the magnesium oxide (MgO), cobalt ferrite (CoFe_2_O_4_) and titanium dioxide (TiO_2_) are dispersed in water. The rising need for highly efficient cooling mechanisms in several sectors and energy-related processes ultimately inspired the current work. The fluid flow and energy propagation is mathematically described in the form of coupled PDEs. The system of PDEs is reduced into non-dimensional forms of ODEs, which are further numerically handled through the Matlab package (bvp4c). It has been observed that the results display that the porosity factor advances the thermal curve, whereas drops the fluid velocity. The effect of heat source/sink raises the energy field. Furthermore, the plate surface illustrates a leading behavior of energy transport over cylinder geometry versus the variation of ternary nanoparticles (NPs). The energy dissemination rate in the cylinder enhances from 4.73 to 11.421%, whereas for the plate, the energy distribution rate boosts from 6.37 to 13.91% as the porosity factor varies from 0.3 to 0.9.

## Introduction

A boundary layer flow occurs close to the surface upon fluid motion across an inclined plane (plate or cylinder). Depending on the Reynolds number, the boundary layer may be laminar or turbulent^1^. Waqas et al.^2^ observed the hybrid nanoliquid flow (HNFs) under the influences of motile microbes, thermal energy, and an electromagnetic field over a stretched inclined plate. Jalali et al.^3^ examined the non-Newtonian dispersion of fluid and thermal exchange in a container with an inclined cylinder. The microscale lattice Boltzmann method is employed to quantitatively analyze the problem. As the temperature-thinning index increases, it can be noticed that a reciprocal correlation between the drag coefficient and Nusselt number. Asjad et al.^4^ observed that CNTs (SWCNTs/MWCNTs) nanomaterials moving over an inclined plate of infinite length were taken in a time-dependent MHD viscoelastic fluid flow using carboxymethyl cellulose also known as CMC, as the base fluid. Utilizing the interaction of impulsive moment with employing Cattaneo-Christov heat flux, the investigation of Powell-Eyring nanofluid motion across an inclined surface at its point of stagnation was conducted by Reddy et al.^5^. Bilal et al.^6^ studied the mixed convection flow of HNFs across an inclined, elongated cylinder using the Darcy-Forchheimer effect. The HNFs were synthesized by combining two different nanoparticles such as GO (graphene oxide) and TiO_2_ (titanium dioxide) including the host fluid. Their results indicated that the HNFs offered the most efficient approach for enhancing heat transfer and could be potentially employed for cryogenic applications. Yusuf et al.^7^ described the impact of porosity parameter, and heat radiation on the rate of entropy generation. Mathur et al.^8^ studied the flow of magneto-Micropolar nanoliquid consisting of TiO_2_ nanoparticles over a flexible surface. The heat transfer of a fluctuating condition in the MHD Casson fluid flow across an inclined layer was investigated by Bharathi et al.^9^. The existing research also takes into account the consequences of the suction/injection, Dufour effects, and magnetic field due to porous media. As the suction parameter increases in all instances of both heating and cooling the porous inclined plate, resulting the velocity declines. Nabwey et al.^10^ concentrated on thermal conduction in the HNFs across an inclined plate affected by chemical reaction and magnetic field. Moreover, the exploration involves the factors of fluctuating reduction or enhancement in thermal radiation and non-homogeneous thermal diffusivity. Pattnaik et al.^11^ reported the free convection flow of gold based nanoliquid across a porous moving wall. Kodi et al.^12^ conducted research on a time-varying hydrological flow across an angled/inclined plate immersed in a penetrable substrate with a chemical reaction and magnetically aligned Soret field. Based on their computations, the presence of an incline angle, magnetization effect, and the Casson fluid constraints exhibit a retarding impact on the velocity. Rasool et al.^13^ used a spectrum relaxation technique to describe the chemical interaction and biological convection process for a slanted magnetized cross nanofluid through an inclined cylinder. Moreover, the information about gyrotactic swimming microbes and fluctuating decrease or increase in thermal radiation is combined. The inclination and orthogonal magnetic influence are examined for each profile. Sudarmozhi et al.^14^ considered the impact of MHD and heat conduction over a perforated inclined vertical plate in addition to the consequences of the radiative boundary layer including Maxwell fluid phenomena. Recent and noteworthy findings concerning such kind of flow have been demonstrated in Refs.^15–21^.

Ternary Hybrid Nanofluids (THNFs) refer to complex fluid suspensions containing three components incorporating a base fluid, two distinct nanoparticle varieties, and additional enhancing agents. These nanofluids combine multiple nanomaterials and additives to achieve synergistic effects, enhancing thermal and transport properties for applications in efficient heat transfer phenomenon, advanced materials, and various engineering fields^22^. In the present study, MgO, CoFe_2_O_4_ and TiO_2_ are used. MgO NPs is utilised in the groundwater and soil remediation, drinking and wastewater treatment, industrial waste due to its acid buffering properties and related efficiency in stabilizing dissolving heavy metal species^23^. CoFe_2_O_4_ NPs have outstanding physical and chemical characteristics and are frequently employed in medicine for imaging, separation by magnetic resonance, drug delivery and biological sensors^24^. TiO_2_ NPs has been extensively utilised since more than one century in a variety of commercial and household goods, such as coatings, paints, glues, plastics, paper, and rubber-based material, dyes for fabric coatings and garments, pottery, ceramics, carpeting, roofing components, beauty products, dental floss, detergent, and filtration^25^. Parida et al.^26^ descried the dynamics of dust nano-particles through the nanofluid. Exploring a new coolant for radiators, Boroomandpour et al.^27^ investigated water-based THNFs comprising nanoparticles with varying shapes, including spherical (Al_2_O_3_), cylindrical (CNT), and Graphene. The proficiency of THNFs nanofluids is expressively effected by the nature, dimension, and mixing proportion of nanomaterials. For a complete knowledge of the hybridization impacts of three-particle nanofluids, it is still necessary to examine a number of topics, such as the impact of mixing various nanoparticle kinds, particle sizes, shapes, and base fluids etc.^28^. Zayan et al.^29^ scrutinized the flow characteristics of THNFs comprising water-based ${\text{Ag}} - {\text{rGO}} - {\text{TiO}}_{{2}}$ and ${\text{Ag}} - {\text{GO}} - {\text{TiO}}_{{2}}$. The solid volume concentration range for all studies was between 0.5 and 0.00005%, with temperatures between 25 and 50 °C. When temperature and shear rates are raised, THNFs viscosity changes by 33% for ${\text{Ag}} - {\text{GO}} - {\text{TiO}}_{{2}}$ and 40% for ${\text{Ag}} - {\text{rGO}} - {\text{TiO}}_{{2}}$. Said et al.^30^ formulated stable nanofluids based on propylene glycol (PG) using zeta potential techniques and scanning electron microscopy were employed to create ${\text{rGO}} - {\text{Fe}}_{{3}} {\text{O}}_{{4}} - {\text{TiO}}_{{2}}$ THNFs. A range of temperatures (25–50 °C) and weight proportions (0.01–0.25 wt%) were employed to investigate density and viscosity variations. At 50 °C, both density and viscosity experienced increments of 2.45% and 133.5%, respectively, for the 0.25 wt% concentration. The assessment of density and viscosity measurements for THNFs produced in the laboratory is faithfully replicated by BRT, ANN, and SVM across an extensive scope of temperatures and ratios of nanoparticle concentration, ultimately leading to a conclusive inference. Acharya^31^ looked into the radiative hybrid nanoliquid flow with the natural convective through a square enclosure. In a boundary layer incorporating metallic nanoparticles (NPs), Alharbi et al.^32^ documented the movement of an electrically conductive incompressible THNFs carrying heat across an elongated cylinder experiencing magnetic induction. Sarada et al.^33^ explored the convective boundary layer flow of a water-based THNFs (CNT-Graphene-Silver) across an irregularly stretched surface. Recently, multiple researchers investigated the THNFs past over various surfaces/geometries under the influence of various nanoparticles Refs.^34–39^.

The exponential heat source/sink has been used by several researchers in their studies. Sajid et al.^40^ scrutinized the dynamics of Maxwell-Sutterby flow across an angled elongating surface with a changeable exponential heat source/sink, variable thermal conductivity, stimulation energy, and MHD. Their computational findings, which are shown here, demonstrate that the temperature profile increases owing to an increase in heat source term. Dawar et al.^41^ discussed the circumstances of 2D electrically conducting MHD flow across an expanding surface in terms of heat source/sink. For a more authentic conclusion, Acharya et al.^42^ enlightened the temperature of chemically reactant nanofluidics motion across an angled spinning plate, considering the immersion of heat source/sink. Mishra et al.^43^ examined the energy transmission with the impact of heat radiation, MHD and uniform heat source. The investigation conducted by Swain et al.^44^ reported the heat transmission of a copper-based nanoliquid dispersed in water over a sheet with nonlinear expansion. Muhammad et al.^45^ studied the 3D MHD flow induced by a varying heat source/sink in heat transfer operations over a flat horizontal surface transporting water-based $GO$ (graphene oxide) nanostructures. The time-varying MHD fluid flow across a porous stretched surface was reported by Mukhtar et al.^46^.

The rising need for highly efficient cooling mechanisms in several sectors and energy-related processes ultimately inspired the current work. Therefore, we have analyzed the steady 2D ternary TNF flow across an inclined permeable cylinder/plate. The exponential heat source/sink, thermal radiation, use of MgO, CoFe_2_O_4_ and TiO_2_ NPS and numerical solution is the main novelty of the proposed model. For the preparation of TNF, the MgO, CoFe_2_O_4_ and TiO_2_ are dispersed in water. The fluid flow and energy propagation is mathematically described in form of coupled PDEs. The system of PDEs is reduced into non-dimensional form of ODEs, which are further numerically handled through the bvp4c. In the pending section, the problem has been verbalized.

## Mathematical formulation

**Figure 1:**
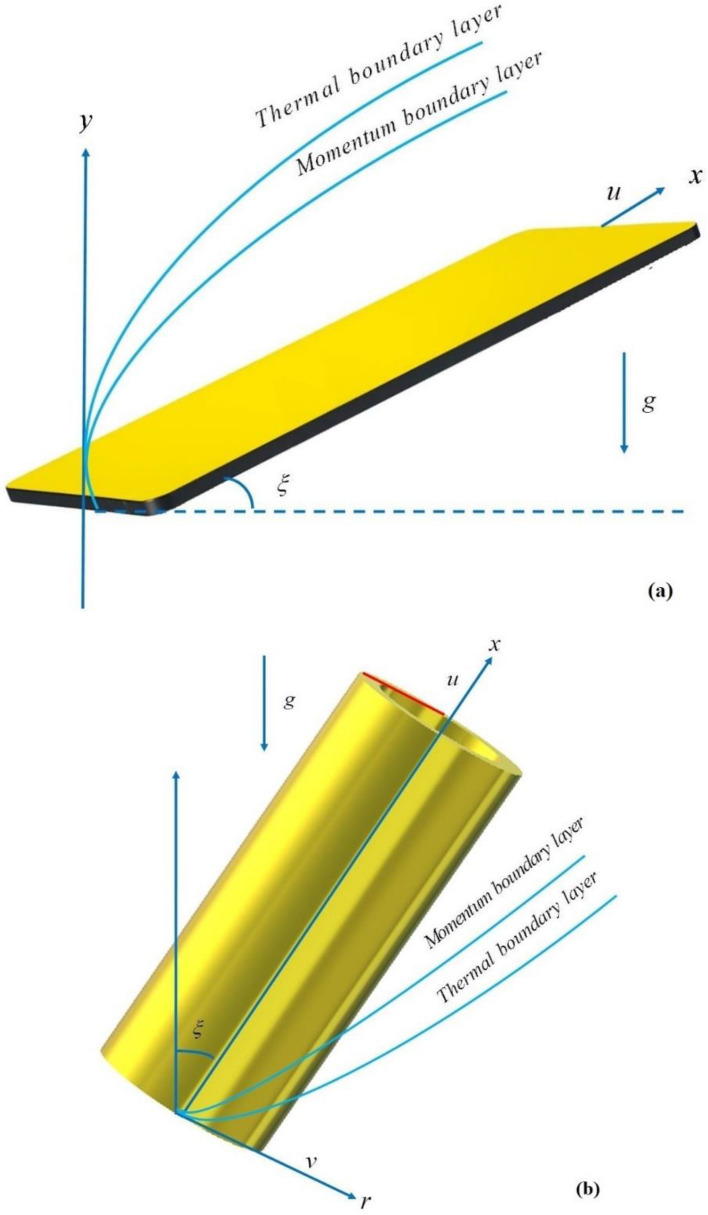
(**a**) Fluid flow over a inclined plate (**b**) Fluid flow across an inclined cylinder.

The steady laminar 2D ternary nanoliquid flow across an inclined permeable cylinder/plate is considered under the consequences of heat source/sink, permeable medium and mixed convection in the present study. For the preparation of TNF, the MgO, CoFe_2_O_4_ and TiO_2_ are dispersed in water. The flow coordinates and physical view is shown in Fig. 1a,b. Here, $x$ and $r$ is the axial and radial coordinates and $u$ signifies the reference velocity. The surface and ambient temperature is signified by $T_{w}$ & $T_{\infty } .$ Furthermore, pressure gradients and external forces are presumed to have no effect on the fluid flow. The modeled equations are expressed as^37^.

1 $$\frac{{\partial \left( {ru} \right)}}{\partial x} + \frac{{\partial \left( {rv} \right)}}{\partial r} = 0,$$

2 $$u\frac{\partial \left( u \right)}{{\partial x}} + v\frac{\partial \left( u \right)}{{\partial r_{{}} }} = v_{Tnf} \left( {\frac{{\partial^{2} u}}{{\partial r_{{}}^{2} }} + \frac{1}{r}\frac{\partial u}{{\partial r}}} \right) + \frac{{\left( {\rho \beta } \right)_{Tnf} g\left( {T_{1} - T_{\infty } } \right)\cos \varsigma }}{{\rho_{Tnf} }} - \frac{{v_{Tnf} }}{{K_{1}^{*} }}u,$$

3 $$u\frac{\partial \left( T \right)}{{\partial x}} + v\frac{\partial \left( T \right)}{{\partial r}} = \alpha_{Tnf} \left( {\frac{{\partial^{2} T}}{{\partial r_{{}}^{2} }} + \frac{1}{{r_{{}} }}\frac{\partial T}{{\partial r}}} \right) - \frac{1}{{\left( {\rho C_{p} } \right)_{nf} }}\frac{\partial }{\partial y}\left( {q_{r} } \right) + \frac{{Q_{e}^{*} }}{{\left( {\rho C_{p} } \right)_{hnf} }}\left( {T - T_{\infty } } \right)exp\left( { - \sqrt {\frac{a}{{\upsilon_{f} }}ny} } \right).$$

The $q_{r}$ term given in Eq. (3) is defined as:

4 $$q_{r} = - \frac{{4\sigma^{*} }}{{3k^{*} }}\frac{\partial }{\partial y}\left( {T^{4} } \right).$$

Here, $\sigma^{*}$ and $q_{r}$ signifies the Stefan-Boltzmann constant and radiative heat flux. By expanding $T^{4}$ centered at $T_{h}$ using Taylor series, we get:

5 $$T^{4} \cong \,4T_{h}^{3} - 3T_{h}^{4} .$$

As a result, the energy equations reformed as:

6 $$\begin{gathered} u\frac{\partial \left( T \right)}{{\partial x}} + v\frac{\partial \left( T \right)}{{\partial r}} = \alpha_{Tnf} \left( {\frac{{\partial^{2} T}}{{\partial r_{{}}^{2} }} + \frac{1}{{r_{{}} }}\frac{\partial T}{{\partial r}}} \right) - \frac{1}{{\left( {\rho C_{p} } \right)_{hnf} }}\frac{{16\sigma^{*} }}{{3k^{*} }}T_{h}^{3} \frac{{\partial^{2} T}}{{\partial y^{2} }} + \frac{{Q_{e}^{*} }}{{\left( {\rho C_{p} } \right)_{hnf} }}\left( {T - T_{\infty } } \right) \hfill \\ exp\left( { - \sqrt {\frac{a}{{\upsilon_{f} }}ny} } \right). \hfill \\ \end{gathered}$$

The boundary conditions (BCs) are^37^:

7 $$\left. \begin{gathered} u = u_{w} = \frac{{U_{0}^{*} x}}{l},\,\,\,v = 0,\,\,\,\,T = T_{w} \,\,\,\,{\text{at}}\,\,\,r = R \hfill \\ u \to 0,\,\,\,\,T \to T_{\infty } ,\,\,\,\,r \to \infty \hfill \\ \end{gathered} \right\}$$

In Eqs. (1), (2) and (3), $$u,\,\,v$$ are the component of velocity along *x* and $$r$$ directions. Whereas $$\mu ,$$ $$\nu = \frac{\mu }{\rho },$$$$\rho ,$$ $$K_{1}^{*} \,\,{\text{and}}\,\,\,\varsigma$$ is the dynamic viscosity, kinematic viscosity, density, surface permeability and an angle of inclination.$$\beta ,\,\,g,\,\,\alpha = \frac{k}{{\rho C_{p} }},\,\,k,\,\,C_{p} \,\,{\text{and}}\,\,\,Q_{1}$$ is the thermal expansion, gravity acceleration, thermal diffusivity, thermal conductivity, specific heat and heat generation and absorption factor.

The similarity variables are^37^:

8 $$\psi = \sqrt {u_{w} v_{f} x} R\,f\left( \eta \right),\,\,\,\,\theta = \frac{{T - T_{\infty } }}{{T_{w} - T_{\infty } }},\,\,\,u = \frac{1}{r}\frac{\partial \psi }{{\partial r}},\,\,\,v = - \frac{1}{r}\frac{\partial \psi }{{\partial x}},\,\,\,\,\eta = \sqrt {\frac{{u_{w} }}{{v_{f} x}}} \left( {\frac{{r_{{}}^{2} - R_{{}}^{2} }}{2R}} \right).$$

By placing Eq. (5) into Eqs. (1), (2), (3) and (4), it is concentrated into the subsequent form:

9 $$\frac{{\left( {\left( {1 + \left( {2\delta_{1} } \right)\eta } \right)f^{\prime\prime\prime} + \left( {2\delta_{1} } \right)f^{\prime\prime}} \right)}}{{B_{1} B_{2} }} - \left( {f^{\prime}} \right)^{2} - \frac{{P_{m} }}{{B_{1} B_{2} }}f^{\prime} + ff^{\prime\prime} + \frac{{B_{3} }}{{B_{2} }}\gamma_{1} \theta \cos \zeta = 0,$$

10 $$\left( {\frac{{k_{Tnf} }}{{k_{f} }} + \frac{4}{3}Rd} \right)\left( {\left( {1 + 2\delta_{1} \eta } \right)\theta^{\prime\prime} + 2\delta_{1} \theta^{\prime}} \right) + Pr\,B_{4} f\theta^{\prime} + Pr\left[ {Q_{e} \exp \left( { - n\eta } \right)} \right].$$

The reduced BCs are:

11 $$\left. \begin{gathered} f\left( \eta \right) = 0,\,\,\,f^{\prime}\left( \eta \right) = 1,\,\,\,\theta \left( \eta \right) = 1\,\,\,\,{\text{at}}\,\,\,\,\eta = 0 \hfill \\ f^{\prime}\left( \infty \right) = 0,\,\,\,\,\theta \left( \infty \right) = 0\,\,\,\,as\,\,\,\,\eta \to \infty \hfill \\ \end{gathered} \right\}$$

In Eqs. (6) & (7), the flow governing constraint are: $\delta_{1}$ is the curvature factor ($\delta_{1} > 0$ signifies cylinder, while $\delta_{1} = 0$ represents plate surface), $P_{m}$ is the porosity factor, $\gamma_{1}$ is the Buoyancy or mixed convection factor, $\zeta$ is the inclined angle, $Pr$ is the Prandtl number, $Q_{e}$ is the heat source/sink constraint, $Gr$ is the local Grashof number and *Rd* is the radiation factor.

$$\begin{gathered} \delta_{1} = \sqrt {\frac{{v_{f} l}}{{U_{0}^{*} R_{{}}^{2} }}} ,\,\,P_{m} = \frac{{v_{f} l}}{{U_{0}^{*} K_{{}}^{*} }},\,\,\gamma_{1} = \frac{Gr}{{Re^{2} }} = \frac{{g\beta \left( {T_{w} - T_{\infty } } \right)l}}{{u_{w} U_{0}^{*} }},\,\,Pr = \frac{{v_{f} }}{{\alpha_{f} }},\,\,\,Rd = \frac{{16\sigma^{*} T_{h}^{3} }}{{3k^{*} k_{f} }},\,\,Q_{e} = \,\frac{{Q_{e}^{*} l}}{{a\left( {\rho C_{p} } \right)_{f} }}, \hfill \\ Gr = \frac{{g\beta \left( {T_{w} - T_{\infty } } \right)z^{3} }}{{v_{f}^{2} }}. \hfill \\ \end{gathered}$$

In Eqs. (6), (7) and (11), *B*_*1*_, *B*_*2*_, *B*_*3*_ and *B*_*4*_ are defined as:

$$\begin{gathered} B_{1} = (1 - \phi_{MgO} )^{2.5} (1 - \phi_{{TiO_{2} }} )^{2.5} (1 - \phi_{{CoFe_{2} O_{4} }} )^{2.5} ,\,\, \hfill \\ B_{2} = \left( {1 - \phi_{{TiO_{2} }} } \right)\left[ {\left( {1 - \phi_{{TiO_{2} }} } \right)\left\{ {\left( {1 - \phi_{{CoFe_{2} O_{4} }} } \right) + \phi_{{CoFe_{2} O_{4} }} \frac{{\rho_{{CoFe_{2} O_{4} }} }}{{\rho_{f} }}} \right\} + \phi_{{TiO_{2} }} \frac{{\rho_{{TiO_{2} }} }}{{\rho_{f} }}} \right] + \phi_{MgO} \frac{{\rho_{MgO} }}{{\rho_{f} }}, \hfill \\ \end{gathered}$$

$$B_{3} = \left( {1 - \phi_{{TiO_{2} }} } \right)\left[ \begin{gathered} \left( {1 - \phi_{{TiO_{2} }} } \right)\left\{ {\left( {1 - \phi_{{CoFe_{2} O_{4} }} } \right) + \phi_{{CoFe_{2} O_{4} }} \frac{{\beta_{{CoFe_{2} O_{4} }} \rho_{{CoFe_{2} O_{4} }} }}{{\beta_{f} \rho_{f} }}} \right\} \hfill \\ + \phi_{{TiO_{2} }} \frac{{\beta_{{TiO_{2} }} \rho_{{TiO_{2} }} }}{{\beta_{f} \rho_{f} }} \hfill \\ \end{gathered} \right] + \phi_{MgO} \frac{{\beta_{MgO} \rho_{MgO} }}{{\beta_{f} \rho_{f} }},$$

$$B_{4} = \left( {1 - \phi_{{TiO_{2} }} } \right)\left[ \begin{gathered} \left( {1 - \phi_{{TiO_{2} }} } \right)\left\{ {\left( {1 - \phi_{{CoFe_{2} O_{4} }} } \right) + \phi_{{CoFe_{2} O_{4} }} \frac{{\left( {Cp} \right)_{{CoFe_{2} O_{4} }} \rho_{{CoFe_{2} O_{4} }} }}{{\left( {Cp} \right)_{f} \rho_{f} }}} \right\} \hfill \\ + \phi_{{TiO_{2} }} \frac{{\left( {Cp} \right)_{{TiO_{2} }} \rho_{{TiO_{2} }} }}{{\left( {Cp} \right)_{f} \rho_{f} }} \hfill \\ \end{gathered} \right] + \phi_{MgO} \frac{{\left( {Cp} \right)_{MgO} \rho_{MgO} }}{{\left( {Cp} \right)_{f} \rho_{f} }}.$$

The experimental values used for above thermophysical properties and their mathematical models are expressed in Tables 1 & 2 as,

**Table 1:** The physical model for trihybrid nanofluid^32^.

Viscosity	$\frac{{\mu_{Tnf} }}{{\mu_{f} }} = \frac{1}{{(1 - \phi_{MgO} )^{2.5} (1 - \phi_{{TiO_{2} }} )^{2.5} (1 - \phi_{{CoFe_{2} O_{4} }} )^{2.5} }},$
Density	$\frac{{\rho_{Tnf} }}{{\rho_{f} }} = \left( {1 - \phi_{{TiO_{2} }} } \right)\left[ {\left( {1 - \phi_{{TiO_{2} }} } \right)\left\{ {\left( {1 - \phi_{{CoFe_{2} O_{4} }} } \right) + \phi_{{CoFe_{2} O_{4} }} \frac{{\rho_{{CoFe_{2} O_{4} }} }}{{\rho_{f} }}} \right\} + \phi_{{TiO_{2} }} \frac{{\rho_{{TiO_{2} }} }}{{\rho_{f} }}} \right] + \phi_{MgO} \frac{{\rho_{MgO} }}{{\rho_{f} }},$
Specific heat	$$\left. {\frac{{(\rho cp)_{Tnf} }}{{\left( {\rho cp} \right)_{f} }} = \phi_{MgO} \frac{{\left( {\rho cp} \right)_{MgO} }}{{\left( {\rho cp} \right)_{f} }} + \left( {1 - \phi_{MgO} } \right)\left[ \begin{gathered} \left( {1 - \phi_{{TiO_{2} }} } \right)\left\{ {\left( {1 - \phi_{{CoFe_{2} O_{4} }} } \right) + \phi_{{CoFe_{2} O_{4} }} \frac{{\left( {\rho cp} \right)_{{CoFe_{2} O_{4} }} }}{{\left( {\rho cp} \right)_{f} }}} \right\} \hfill \\ + \phi_{{TiO_{2} }} \frac{{\left( {\rho cp} \right)_{{TiO_{2} }} }}{{\left( {\rho cp} \right)_{f} }} \hfill \\ \end{gathered} \right]} \right\}$$
Thermal conduction	$$\left. \begin{gathered} \frac{{k_{Tnf} }}{{k_{hnf} }} = \left( {\frac{{k_{{CoFe_{2} O_{4} }} + 2k_{hnf} - 2\phi_{{CoFe_{2} O_{4} }} \left( {k_{hnf} - k_{{CoFe_{2} O_{4} }} } \right)}}{{k_{{CoFe_{2} O_{4} }} + 2k_{hnf} + \phi_{{CoFe_{2} O_{4} }} \left( {k_{hnf} - k_{{CoFe_{2} O_{4} }} } \right)}}} \right),\frac{{k_{hnf} }}{{k_{nf} }} = \left( {\frac{{k_{{TiO_{2} }} + 2k_{nf} - 2\phi_{{TiO_{2} }} \left( {k_{nf} - k_{{TiO_{2} }} } \right)}}{{k_{{TiO_{2} }} + 2k_{nf} + \phi_{{TiO_{2} }} \left( {k_{nf} - k_{{TiO_{2} }} } \right)}}} \right), \hfill \\ \frac{{k_{nf} }}{{k_{f} }} = \left( {\frac{{k_{MgO} + 2k_{f} - 2\phi_{MgO} \left( {k_{f} - k_{MgO} } \right)}}{{k_{MgO} + 2k_{f} + \phi_{MgO} \left( {k_{f} - k_{MgO} } \right)}}} \right), \hfill \\ \end{gathered} \right\}$$
Electrical conductivity	$$\left. \begin{gathered} \frac{{\sigma_{Tnf} }}{{\sigma_{hnf} }} = \left[ {1 + \frac{{3\left( {\frac{{\sigma_{{CoFe_{2} O_{4} }} }}{{\sigma_{hnf} }} - 1} \right)\phi_{{CoFe_{2} O_{4} }} }}{{\left( {\frac{{\sigma_{{CoFe_{2} O_{4} }} }}{{\sigma_{hnf} }} + 2} \right) - \left( {\frac{{\sigma_{{CoFe_{2} O_{4} }} }}{{\sigma_{hnf} }} - 1} \right)\phi_{{CoFe_{2} O_{4} }} }}} \right],\,\frac{{\sigma_{hnf} }}{{\sigma_{nf} }} = \left[ {1 + \frac{{3\left( {\frac{{\sigma_{{TiO_{2} }} }}{{\sigma_{nf} }} - 1} \right)\phi_{{TiO_{2} }} }}{{\left( {\frac{{\sigma_{{TiO_{2} }} }}{{\sigma_{nf} }} + 2} \right) - \left( {\frac{{\sigma_{{TiO_{2} }} }}{{\sigma_{nf} }} - 1} \right)\phi_{{TiO_{2} }} }}} \right], \hfill \\ \,\,\,\frac{{\sigma_{nf} }}{{\sigma_{f} }} = \left[ {1 + \frac{{3\left( {\frac{{\sigma_{MgO} }}{{\sigma_{f} }} - 1} \right)\phi_{MgO} }}{{\left( {\frac{{\sigma_{MgO} }}{{\sigma_{f} }} + 2} \right) - \left( {\frac{{\sigma_{MgO} }}{{\sigma_{f} }} - 1} \right)\phi_{MgO} }}} \right] \hfill \\ \end{gathered} \right\}$$

**Table 2:** The experimental values of MgO, CoFe_2_O_4_, TiO_2_ and water^32^.

Base fluid & nanoparticles	$\rho (kg/m^{3} )$	$Cp(J/kgK)$	$k(W/mK)$	$\beta \times 10^{5} \left( {K^{ - 1} } \right)$	$\sigma (S/m)$
Cobalt ferrite CoFe_2_O_4_	4907	700	3.7	**–**	$5.51 \times 10^{9}$
Titanium dioxide TiO_2_	4250	686.2	8.9538	0.9	$2.38 \times 10^{6}$
Magnesium oxide MgO	3560	955	45	1.26	$1.42 \times 10^{ - 3}$
Pure water H_2_O	997.1	4179	0.613	21	0.05

For engineering coefficients and their reduced form:

12 $$\begin{gathered} C_{f} = \frac{{\mu_{Tnf} }}{{\rho_{f} uw^{2} }}\left. {\frac{\partial u}{{\partial r}}} \right|_{r = R} ,\,\,\,\,\,\,\,N_{u} = \frac{{ - xk_{Tnf} }}{{k_{f} \left( {T_{w} - T_{\infty } } \right)}}\left. {\frac{\partial T}{{\partial r}}} \right|_{r = R} , \hfill \\ \hfill \\ \sqrt {Re} \,C_{f} = \frac{1}{{B_{1} }}f^{\prime\prime}\left( 0 \right),\,\,\,\frac{{N_{u} }}{{\sqrt {Re\,} }} = - \left( {\frac{{k_{Tnf} }}{{k_{f} }} + \frac{4}{3}Rd} \right)\theta^{\prime}\left( 0 \right). \hfill \\ \end{gathered}$$

Here *Re* is the local Reynold number.

## Numerical solution and validation

The numerical simulation of the system of ODEs (Eqs. 9, 10 and 11) that characterize the fluid flow and heat transportation is discoursed in this segment. The results are achieved using the MATLAB built-in package bvp4c. The bvp4c code is based on the three-stage Lobatto III formula. The collocation polynomials give accuracy up to fourth-order.

The system of coupled ODEs Eqs. (9) & (10) and (11) are reduced further to the 1st order by using the variables as:

13 $$\rlap{--} \mathchar'26\mkern-10mu\lambda _{1} (\eta ) = f,\,\,\,\,\rlap{--} \mathchar'26\mkern-10mu\lambda _{2} (\eta ) = f^{\prime},\,\,\,\,\rlap{--} \mathchar'26\mkern-10mu\lambda _{3} (\eta ) = f^{\prime\prime},\,\,\,\rlap{--} \mathchar'26\mkern-10mu\lambda _{4} = \theta (\eta ),\,\,\,\,\rlap{--} \mathchar'26\mkern-10mu\lambda _{5} = \theta ^{\prime}(\eta ).$$

By inserting Eq. (13) in Eqs. (9), (10) & (11), we get:

14 $$\begin{gathered} \frac{{\left( {\left( {1 + \left( {2\delta _{1} } \right)\eta } \right)\rlap{--} \mathchar'26\mkern-10mu\lambda ^{\prime}_{3} \left( \eta \right) + \left( {2\delta _{1} } \right)\rlap{--} \mathchar'26\mkern-10mu\lambda _{3} \left( \eta \right)} \right)}}{{B_{1} B_{2} }} - \left( {\rlap{--} \mathchar'26\mkern-10mu\lambda _{2} \left( \eta \right)} \right)^{2} - \frac{{P_{m} }}{{B_{1} B_{2} }}\rlap{--} \mathchar'26\mkern-10mu\lambda _{2} \left( \eta \right) + \rlap{--} \mathchar'26\mkern-10mu\lambda _{1} \left( \eta \right)\rlap{--} \mathchar'26\mkern-10mu\lambda _{3} \left( \eta \right) \hfill \\ + \frac{{B_{3} }}{{B_{2} }}\gamma _{1} \theta \cos \zeta = 0, \hfill \\ \end{gathered}$$

15 $$\left( {\frac{{k_{{Tnf}} }}{{k_{f} }} + \frac{4}{3}Rd} \right)\left( {\left( {1 + 2\delta _{1} \eta } \right)\rlap{--} \mathchar'26\mkern-10mu\lambda ^{\prime}_{5} \left( \eta \right) + 2\delta _{1} \rlap{--} \mathchar'26\mkern-10mu\lambda _{5} \left( \eta \right)} \right) + \Pr \,B_{4} \rlap{--} \mathchar'26\mkern-10mu\lambda _{1} \left( \eta \right)\rlap{--} \mathchar'26\mkern-10mu\lambda _{5} \left( \eta \right) + \Pr \left[ {Q_{e} \exp \left( { - n\eta } \right)} \right].$$

The reduced BCs are:

16 $$\left. \begin{gathered} \rlap{--} \mathchar'26\mkern-10mu\lambda _{1} \left( \eta \right) = 0,\,\,\,\rlap{--} \mathchar'26\mkern-10mu\lambda _{2} \left( \eta \right) = 1,\,\,\,\rlap{--} \mathchar'26\mkern-10mu\lambda _{4} \left( \eta \right) = 1\,\,\,\,at\,\,\,\,\eta = 0 \hfill \\ \rlap{--} \mathchar'26\mkern-10mu\lambda _{2} \left( \infty \right) = 0,\,\,\,\,\rlap{--} \mathchar'26\mkern-10mu\lambda _{4} \left( \infty \right) = 0\,\,\,\,as\,\,\,\,\eta \to \infty \hfill \\ \end{gathered} \right\}$$

### Validation

For the validity of results obtained via bvp4c is statistically equated to published study through Table 3, which expose that the present results are reliable and accurate.

**Table 3:** The validation of the current results against the published literature for $- \left( {f^{\prime\prime}\left( 0 \right)} \right)$.

*P*_*m*_	Madhukesh et al.^37^,$- \left( {f^{\prime\prime}\left( 0 \right)} \right)$			Present work,$- \left( {f^{\prime\prime}\left( 0 \right)} \right)$	
	SRM	Analytical	RK-45	bvp4c	Error
1.0	1.414313567	1.41431357	1.4143376	1.41433782	0.001029
2.0	1.73215082	1.73215082	1.7321518	1.73215193	0.001035
3.0	2.44958975	2.44958975	2.4495898	2.44958994	0.002069
4.0	3.31672480	3.31672480	3.3167248	3.31672499	0.001745

## Results and discussion

**Figure 2:**
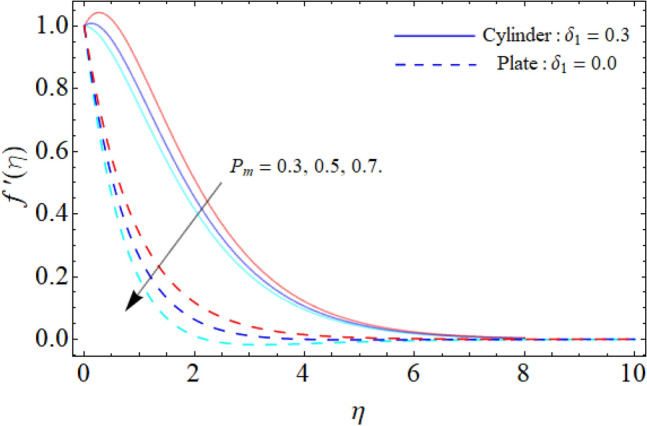
Porosity factor versus $f^{\prime}\left( \eta \right).$

**Figure 3:**
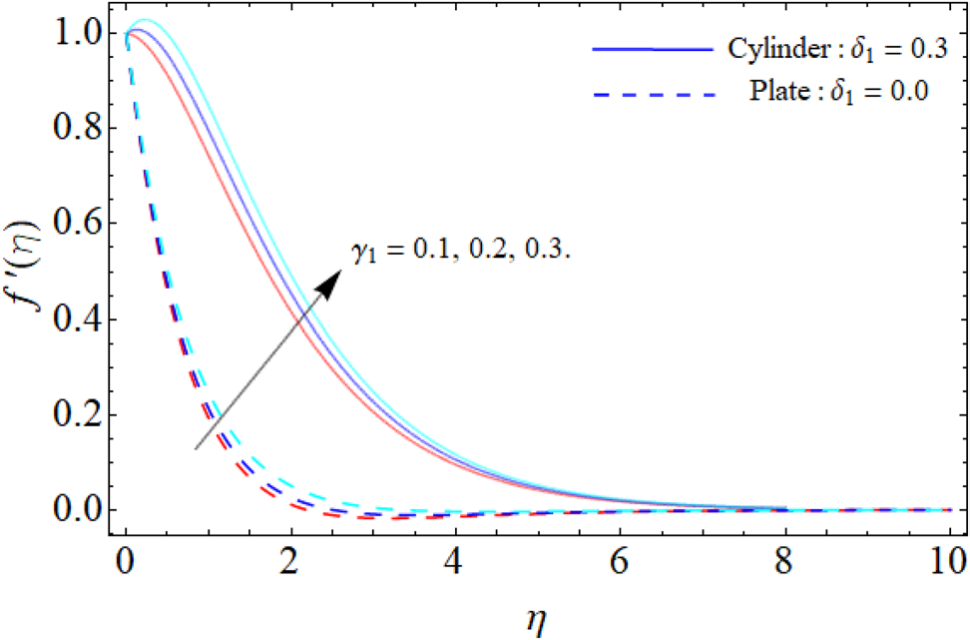
Mixed convection or Buoyancy factor versus $f^{\prime}\left( \eta \right).$

In this segment, we elucidate the physical mechanism behind the graphical and tabular results and their variation versus different physical flow constraints. The steady two-dimension 2D ternary nanoliquid flow under the consequences of heat source/sink, permeable medium and mixed convection across an inclined permeable cylinder/plate is analyzed in this study. MgO, CoFe_2_O_4_, and TiO_2_ are dissolved in water to synthesize the TNF. The fluid flow and energy propagation is mathematically described in form of coupled PDEs. The system of PDEs is reduced into non-dimensional form of ODEs, which are further numerically handled through bvp4c. The detail analysis is presented as:

Figure 2 discloses the impact of porosity factor $\left( {P_{m} } \right)$ on the fluid velocity $f^{\prime}\left( \eta \right).$ It can be observed that the fluid flow drops with the rising porosity of the plate or cylinder surface. Physically, the rising numbers of pores over the surface, suck the fluid particles, which resist to the fluid flow and causes reduction in the velocity boundary layer. Figure 3 shows the impact of mixed convection/buoyancy factor $\left( {\gamma_{1} } \right)$ on $f^{\prime}\left( \eta \right).$ It can be seen that the effect of buoyancy factor enhances the flow velocity. Physically, more significant values of mixed convection factor $\left( {\gamma_{1} } \right)$ demonstrate that the thermal buoyancy force is more effective on the fluid flow. The fluid's velocity profile rises as a consequence of the thermal buoyancy force's dominance. It functions in opposition to the flow direction. Greater thermal buoyancy forces push fluid movement, which causes an increase in fluid velocity and a lowering of temperature.

**Figure 4:**
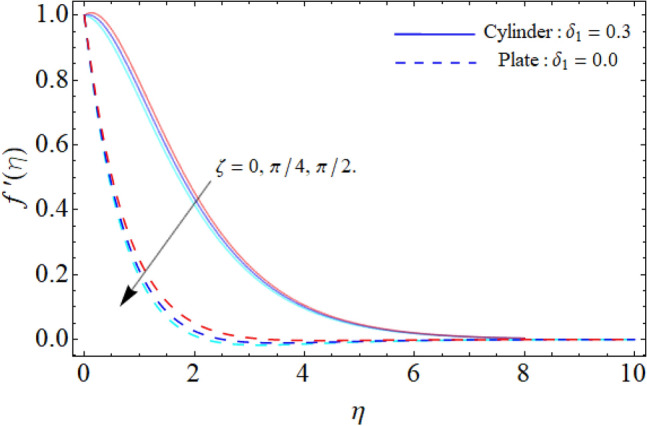
Inclination angle versus $f^{\prime}\left( \eta \right).$

**Figure 5:**
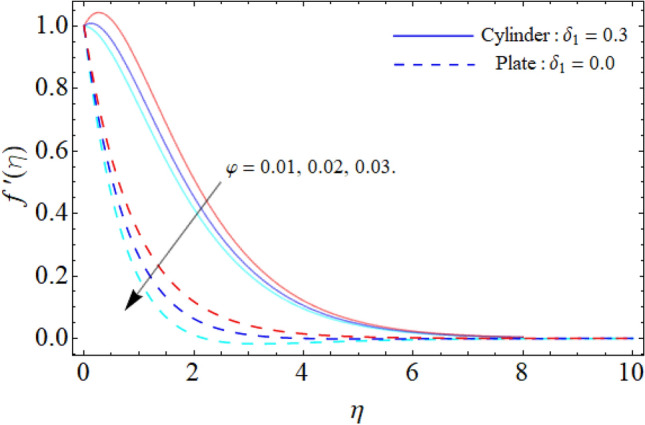
Ternary nanoparticle versus $f^{\prime}\left( \eta \right).$

Figure 4 discloses the upshot of inclination angle versus $f^{\prime}\left( \eta \right).$ It can be perceived that rising angle of inclination of plate and cylinder from $0^{ \circ }$ to $90^{ \circ }$ drops the flow velocity. Fluid velocity reduces as the angle of inclination rises because the buoyancy force has less impact on the fluid flow. This reduction in fluid velocity improves thermal efficiency by improving heat transportation. In industries where proper thermal management is essential, such as thermal exchangers, electronic gadgets and cooling mechanisms, this effect is especially pertinent. It is possible to improve the entire system's effectiveness and optimize the thermal performance by adjusting the angle of inclination of the flow system. Furthermore, the plate has a greater temperature circulation than cylinder. Figure 5 exposes the impact of ternary nanoparticle on the $f^{\prime}\left( \eta \right).$ It has been observed that the fluid flow falloffs with the rising numbers of ternary NPs. Physically, the density of MgO, CoFe_2_O_4_ and TiO_2_-NPs is higher than the density of water, that ‘why, the accumulation of these NPs in water falloffs the fluid velocity.

**Figure 6:**
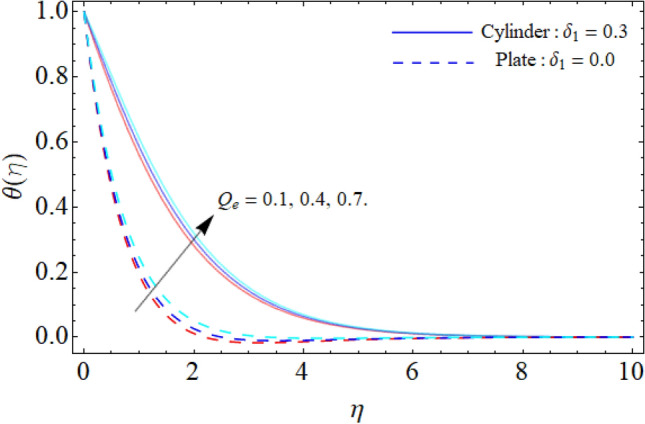
Heat source/sink versus $\theta \left( \eta \right).$

**Figure 7:**
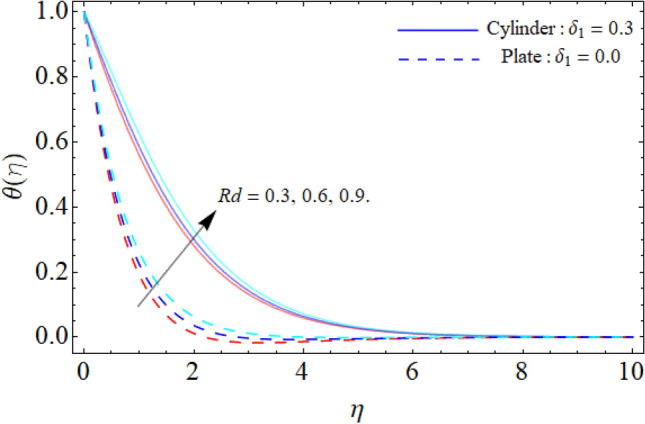
Thermal radiation factor versus $\theta \left( \eta \right).$

**Figure 8:**
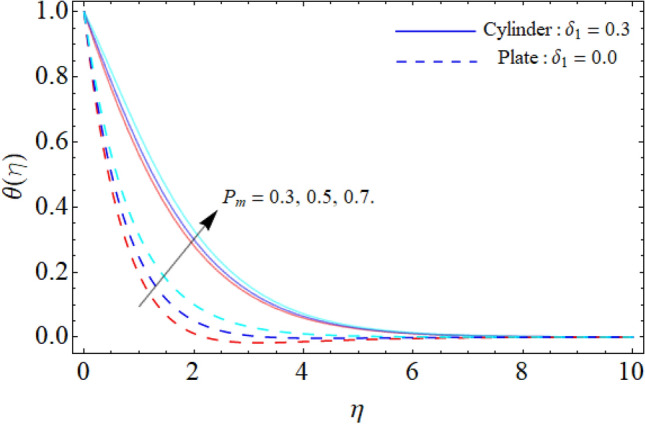
Porosity factor versus $\theta \left( \eta \right).$

Figures 6 and 7 reveal the impact of heat source/sink and thermal radiation factor on the $\theta \left( \eta \right).$ Physically, the rising effect of *Q*_*e*_ and *Rd* both boost the energy profile of TNF. Because, the implementation of both parameters provides an additional heat to the fluid, which results in the advancement of energy curve. Figure 8 divulges the impact of porosity factor $\left( {P_{m} } \right)$ on the energy field $\theta \left( \eta \right).$ It can be detected that the fluid temperature enriches with the rising porosity of the plate or cylinder surface. Physically, the rising numbers of pores over the surface, suck the fluid particles, which resist to the fluid flow and cause advancement in the thermal profile.

**Figure 9:**
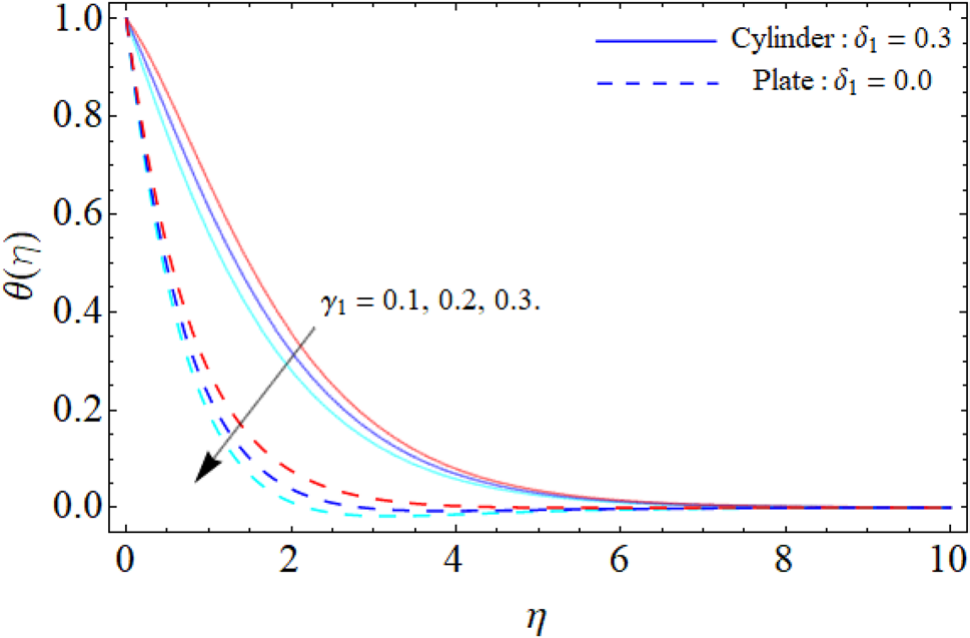
Mixed convection or Buoyancy factor versus $\theta \left( \eta \right).$

**Figure 10:**
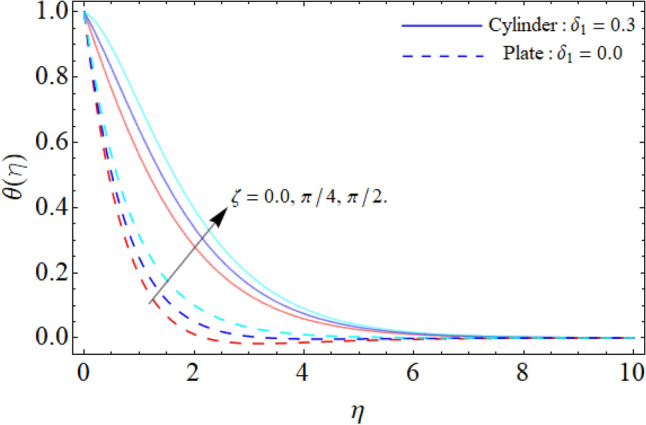
Inclination angle versus $\theta \left( \eta \right).$

**Figure 11:**
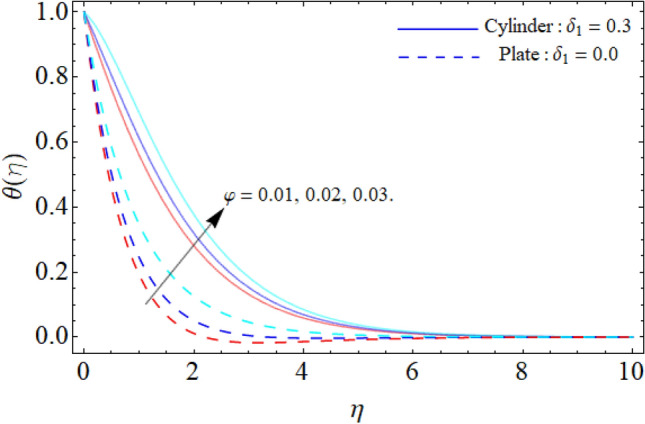
Ternary nanoparticle versus $\theta \left( \eta \right).$

**Figure 12:**
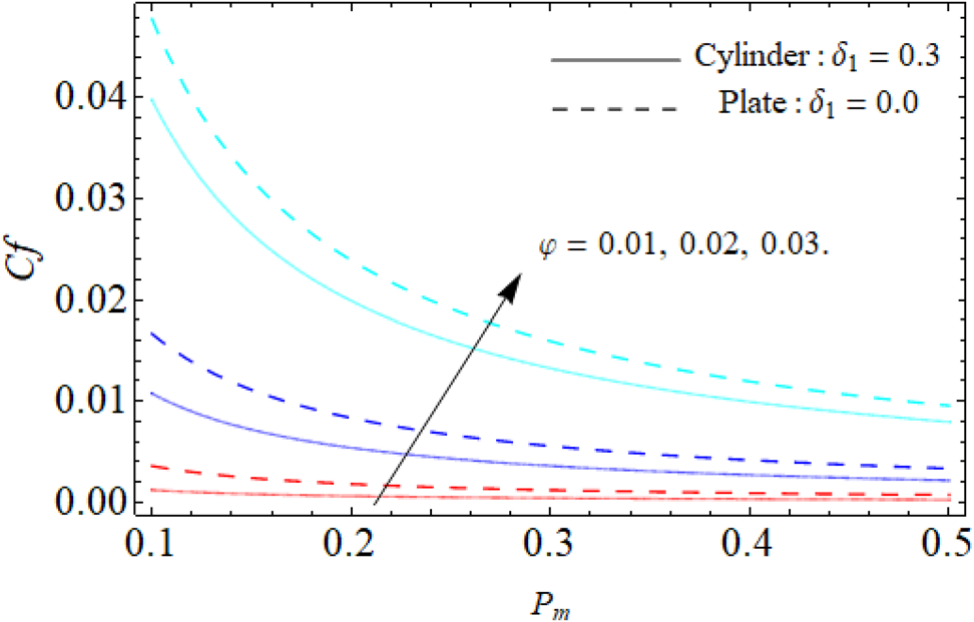
Skin fraction (*Cf*) versus the variation of ternary nanoparticle.

**Figure 13:**
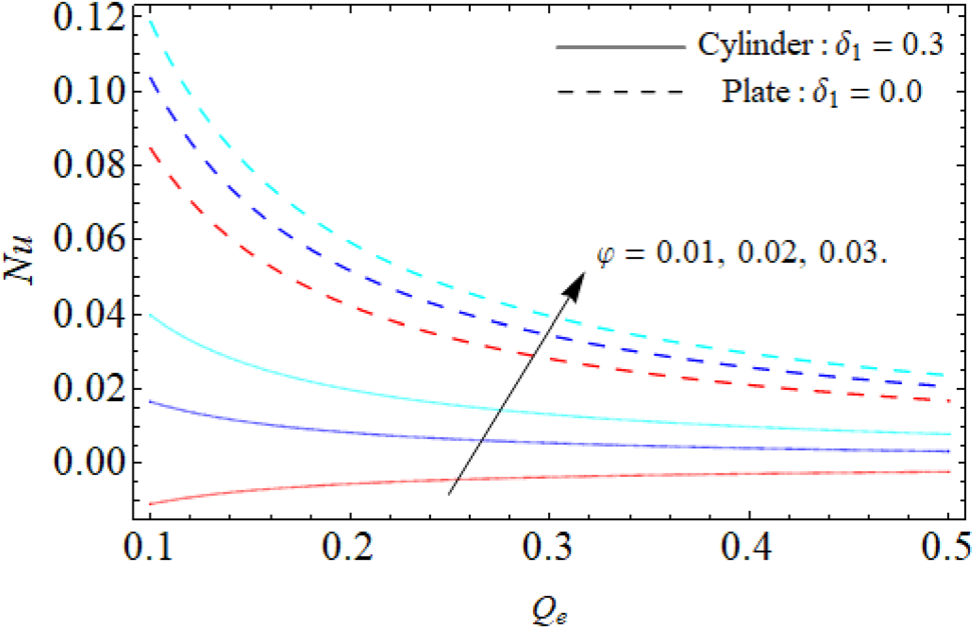
Nusselt number (*Nu*) versus the variation of ternary nanoparticle.

Figure 9 displays the impact of mixed convection/buoyancy factor $\left( {\gamma_{1} } \right)$ on $\theta \left( \eta \right).$ It can be seen that the effect of buoyancy factor falloffs the energy field. Physically, more significant values of mixed convection factor $\left( {\gamma_{1} } \right)$ demonstrate that the thermal buoyancy force is more effective on the fluid flow. The fluid's velocity profile rises as a consequence of the thermal buoyancy force’s dominance. Greater thermal buoyancy forces push fluid movement, which causes an increase in fluid velocity and a lowering of temperature $\theta \left( \eta \right).$ Figure 10 exposes that the thermal profiles of ternary nanoliquid rises with the upshot of Inclination angle. Figure 11 disclosures the influence of ternary nanoparticle on $\theta \left( \eta \right).$ It has been detected that the fluid thermal profile declines with the rising quantities of ternary NPs. Physically, the density of MgO, CoFe_2_O_4_ and TiO_2_-NPs is higher than the density of water, that ‘why, the accumulation of these NPs in water enhances the viscosity of the fluid. This can absorb more heat and results in the shrinking of energy field. Figures 12 and 13 illustrates the graphical results of Skin fraction and Nusselt number. It can be observed that the velocity and energy transportation rate of ternary nanoliquid accelerates with the variation of MgO, CoFe_2_O_4_ and TiO_2_-NPs. This property of ternary nanoliquid is more significant for the industrial and engineering uses.

**Figure 14:**
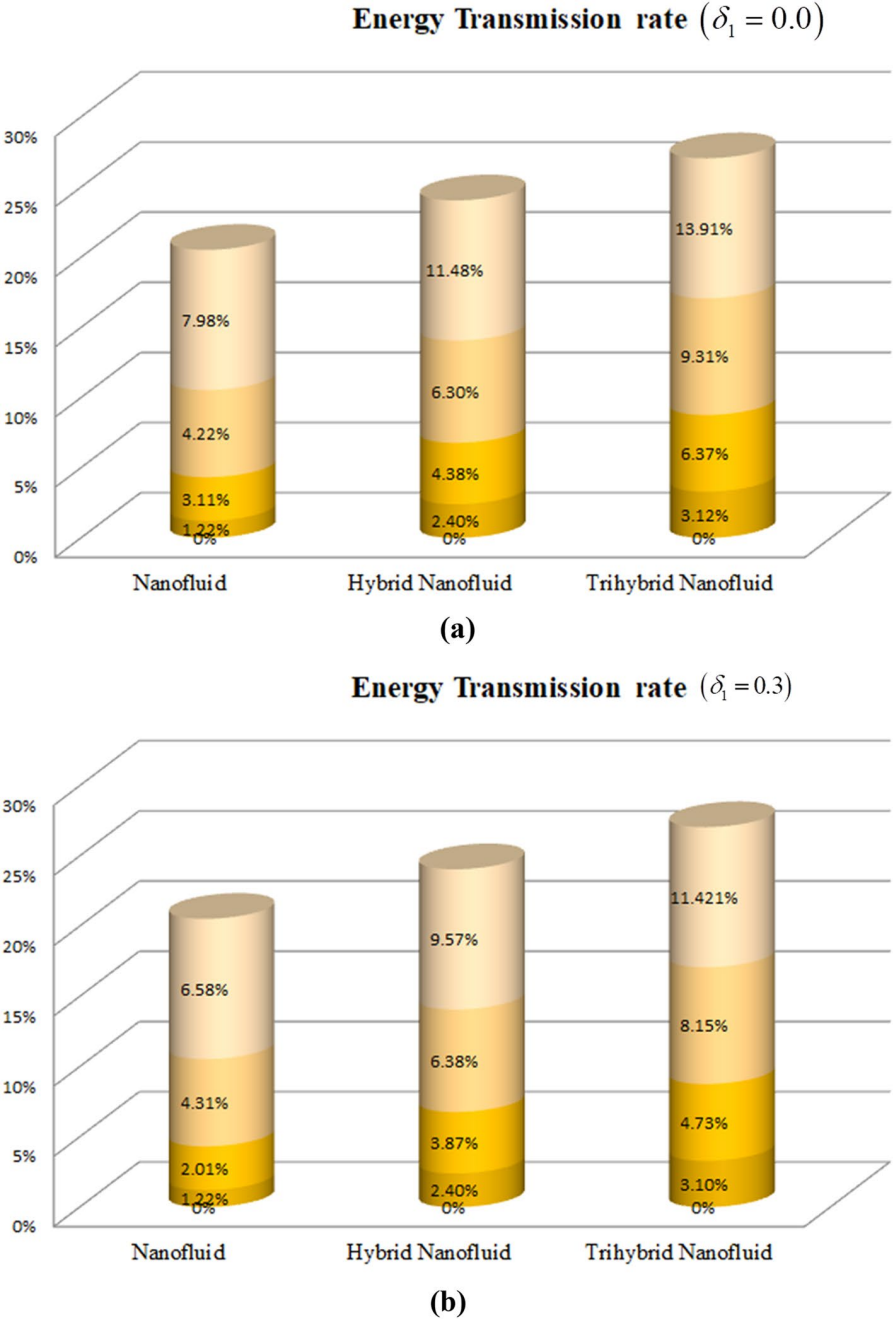
(**a**) Energy transfer rate for plate (**b**) Energy transfer rate for cylinder.

Figure 14a,b reveal the energy transfer rate for Plate and Cylinder versus the varying values of ternary nanoparticles $\left( {\varphi = 0.0,\,\,0.01,\,\,0.02,\,\,0.03,\,\,0.04} \right)$. It can be clearly perceived that the energy propagation rate in case of plate is higher than the cylinder. Here $\varphi$ is considered as $\varphi = \phi_{1} ,$ $\varphi = \phi_{2}$ and $\varphi = \phi_{3} .$

Table 4 shows the percentage % values of cylinder $\left( {\delta_{1} = 0.3} \right)$ and plate $\left( {\delta_{1} = 0.0} \right)$ for the energy transmission rate. It can concluded form both the cases that the varying impact of thermal radiation and heat source/sink factor remarkably boost the energy transfer rate.

**Table 4:** Percentage % values of $Nu\,$ for cylinder $\left( {\delta_{1} = 0.3} \right)$ and plate $\left( {\delta_{1} = 0.0} \right)$.

Parameters				Cylinder (MgO/water),$\left( {\delta_{1} = 0.3} \right)$	Cylinder (MgO-TiO_2_/water),$\left( {\delta_{1} = 0.3} \right)$	Cylinder (MgO-TiO_2_–CoFe_2_O_4_/water),$\left( {\delta_{1} = 0.3} \right)$	Plate (MgO-TiO_2_–CoFe_2_O_4_/water),$\left( {\delta_{1} = 0.0} \right)$
$P_{m}$	$\gamma_{1}$	$\zeta$	$Q_{e}$	Percentage %	Percentage %	Percentage %	Percentage %
0.3	0.1	$30^{ \circ }$	0.1	2.05%	5.9%	11.76%	15.89%
0.5				2.07%	5.03%	11.02%	15.04%
0.7				2.21%	5.32%	11.45%	15.56%
0.3	0.1			2.07%	5.09%	11.13%	15.24%
	0.2			2.02%	5.04%	11.37%	15.56%
	0.3			1.87%	4.89%	10.56%	14.83%
	0.1	$0^{ \circ }$		2.07%	5.16%	11.21%	15.89%
		$30^{ \circ }$		2.07%	3.25%	9.74%	13.23%
		$60^{ \circ }$		2.08%	5.18%	9.98%	13.53%
		$30^{ \circ }$	0.1	0.13%	3.25%	8.43%	12.91%
			0.4	0.16%	3.31%	8.57%	12.92%
0.3	0.1	$30^{ \circ }$	0.7	0.17%	3.42%	8.72%	12.98%

## Conclusions

We have assessed the steady the 2D ternary nanofluid flow across an inclined permeable cylinder/plate. The TNF flow has been examined under the consequences of heat source/sink, permeable medium and mixed convection. MgO, CoFe_2_O_4_, and TiO_2_ are dissolved in water to synthesize the TNF. The fluid flow and energy propagation is mathematically described in form of coupled PDEs. The system of PDEs is reduced into non-dimensional form of ODEs, which are further numerically handled through bvp4c. The core deductions are:

- The plate surface illustrates a leading behavior of energy transport over cylinder geometry versus the variation of ternary nanoparticles (NPs).
- The fluid flow drops with the rising porosity of the plate or cylinder surface, whereas the effect of buoyancy factor enhances the flow velocity.
- The rising angle of inclination of plate and cylinder from $0^{ \circ }$ to $90^{ \circ }$ drops the flow velocity, whereas enhances the energy profile.
- The thermal profile and fluid flow declines with the rising numbers of MgO, CoFe_2_O_4_ and TiO_2_-NPs.
- The energy dissemination rate in the cylinder enhances from 4.73 to 11.421%, whereas for the plate, energy distribution rate boost form 6.37 to 13.91% as the porosity factor varies from 0.3 to 0.7.
- The rising effect of heat source/sink, porous surface parameter and thermal radiation factor boost the energy profile of TNF.
- The fluid temperature enriches with the rising porosity of the plate or cylinder surface. Physically, the rising numbers of pores over the surface, suck the fluid particles, which resist to the fluid flow and cause advancement in the thermal profile.
- The velocity and energy transportation rate of ternary nanoliquid accelerates with the variation of MgO, CoFe_2_O_4_ and TiO_2_-NPs. This property of ternary nanoliquid is more significant for the industrial and engineering uses.
- The present model can be modified to other sort of fluid models and can be solved by other numerical and analytical techniques. It can also be further extended by using different geometry.

## Data Availability

All data used in this manuscript have been presented within the article.

## Abbreviations

$u,\,\,v$: Component of velocity
$$\nu$$: Kinematic viscosity
$$K_{1}^{*}$$: Surface permeability
$$\beta$$: Thermal expansion
$Q_{1}$: Heat source factor
$$T_{w}$$: Surface temperature
$$P_{m}$$: Porosity factor
$$g$$: Gravity acceleration
$\gamma_{1}$: Mixed convection factor
$$\alpha$$: Electrical conductivity
$Q_{e}$: Heat source/sink constraint
$Gr$: Grash of number
TiO_2_: Titanium dioxide
NPs: Nanoparticles
CoFe_2_O_4_: Cobalt ferrite
$\sigma^{*}$: Stefan-Boltzmann constant
$$\mu ,$$: Dynamic viscosity
$q_{r}$: Radiative heat flux
$$C_{p}$$: Specific heat
$$\rho$$: Density
$\varsigma$: Angle of inclination
$$\delta_{1}$$: Curvature factor
$$T_{\infty } .$$: Ambient temperature
$$\delta_{1} > 0$$: Cylinder surface
$$k$$: Thermal conductivity
$$\delta_{1} = 0$$: Plate surface
$$Pr$$: Prandtl number
*Rd*: Radiation factor
bvp4c: Matlab package
MgO: Magnesium oxide
*C*_*f*_: Skin friction
